# Analytical sensitivity of a multiplex quantitative PCR for *Toxoplasma gondii* and *Neospora caninum*

**DOI:** 10.1007/s00436-023-07796-5

**Published:** 2023-02-18

**Authors:** Marcus Truong, Jan Šlapeta

**Affiliations:** 1 Sydney School of Veterinary Science, Faculty of Science, The University of Sydney, Sydney, NSW 2006 Australia; 2 Institute for Infectious Diseases, The University of Sydney, University of Sydney, Sydney, NSW 2006 Australia

**Keywords:** Cyst-forming coccidia, Real-time PCR, Diagnostics, DNA, Neosporosis, Toxoplasmosis, *Hammondia*

## Abstract

Cyst-forming coccidia, *Toxoplasma gondii* and *Neospora caninum*, are recognised as important causes of animal disease. Molecular diagnostics based on the presence of DNA in animal tissue are required to specifically detect *T. gondii* and *N. caninum* while achieving high levels of analytical sensitivity. We optimised available single-plex probe base qPCR assays into a multiplexed qPCR panel to detect cyst-forming coccidia, i.e. *T. gondii* and *N. caninum*. The *T. gondii* assay is based on a 529-bp repetitive (REP) element and the *N. caninum* assay on the NC5 repetitive region. Using target sequence synthetic DNA, the limit of detection (LOD) was determined to be 100 copies, that is less than a single tachyzoite of either *T. gondii* or *N. caninum*. The *T. gondii* and *N. caninum* multiplexed qPCR assay optimised in this study can be used to effectively detect parasite DNA for diagnostic purposes in animal tissue.

## Introduction

Cyst-forming coccidia, such as *Toxoplasma gondii* and *Neospora caninum*, are recognised as important causes of animal and human disease (Dubey 2021; Dubey et al. 2017). Molecular diagnostics are based on the presence of parasite DNA in animal tissue. The differentiation of *T. gondii* and *N. caninum* is of utmost importance due to the clinical symptoms associated with toxoplasmosis and neosporosis. For this reason, PCR techniques based on repetitive regions found in the genomes of *T. gondii* and *N. caninum* have historically been selected as targets for amplification (Muller et al. 1996). Two *T. gondii* DNA markers commonly targeted by PCR-based diagnostics are the B1 gene and 529-bp repetitive (REP) element (Homan et al. 2000). For *N. caninum*, the NC5 region has become the standard target because of its multicopy nature (Muller et al. 1996). As technology progressed, conventional PCRs were replaced by quantitative PCR (qPCR) methods. qPCR allowed for the rapid and specific detection of DNA in organisms such as *T. gondii* and *N. caninum* (Fekkar et al. 2008; Ghalmi et al. 2008). Alongside *T. gondii* and *N. caninum*, animals can also be infected with *Hammondia* spp., an organism that is often considered non-pathogenic, yet some case reports suggest otherwise (Allan et al. 2022; Reichel et al. 2007; Schares et al. 2021; Šlapeta et al. 2002; Steffl and Nautscher 2019). A highly specific qPCR assay for *Hammondia hammondi*, based on the repetitive element Hhamm222, has recently been developed (Schares et al. 2021).

The aim of this study was to optimise and evaluate the performance of available single-plex probe-based qPCR assays into a multiplexed qPCR panel to detect cyst-forming coccidia, i.e. *T. gondii* and *N. caninum* in host tissue or fluid samples during suspected clinical disease.

## Materials and methods

This study used the synthetic *T. gondii* DNA fragments B1 (AF179871) and REP (AF146527) (243 nt), a *H. hammondi* DNA fragment Hham222 (KC223619), and an *N. caninum* DNA fragment NC5 (X84238) targets (232nt). The synthetic DNA was obtained as gBlocks (Integrated DNA Technologies, Australia). The synthetic DNA was resuspended in distilled water and a stock solution of 10^9^ copies/μL was made and stored at – 20 °C in clear plastic microtubes. Stock dilutions were discarded and made fresh after five thaw-freeze cycles.

Four probe-based qPCR assays were used throughout this study targeting *T. gondii*, *H. hammondi*, and *N. caninum* (Table 1). The qPCR assays were adopted from published studies (Ghalmi et al. 2008; Schares et al. 2021). In addition, probe-based qPCRs targeting the partial canine glyceraldehyde 3-phosphate dehydrogenase (GAPDH) region were performed to verify the presence of mammalian DNA (Table 1). All probes and primers were obtained from Integrated DNA Technologies (IDT, Australia). The Myra Liquid Handling System (Bio Molecular Systems, Australia) was used to prepare and multiplex all PCR reactions. All qPCRs were run in 10 μl volume with 1 μl of template (10^6^ to 1 synthetic DNA template copy per reaction), 5 μl of Luna® Universal Probe qPCR Master Mix (New England Biolabs, Australia), 400 nM of forward and reverse primers, and 100 nM of the appropriate probes. The cycling conditions for all runs were 95 °C for 3 min as initial denaturation followed by 40 cycles of 95 °C for 5 s and 60 °C for 15 s. All qPCRs were run using a CFX96 (BioRad, Australia) with runs analysed using CFX Maestro 3.2 (BioRad, Australia) to construct standard curves from serial dilutions and obtained efficacy (*E*) and *R*^2^ values. Cycle threshold (*C*_t_) was auto-calculated. Assays were considered optimised if *E* = 90 to 110% and *R*^2^ > 0.94. This study defines analytical sensitivity as the limit of detection (LOD) being the lowest concentration of DNA (in copy numbers) detected in all three replicates. The assays were either run with respective synthetic controls only or further spiked with mammalian DNA (100 ng).

**Table 1:** Primers and probes to amplify cyst-forming coccidia and mammalian DNA

Toxoplasma gondii B1 assay	
^1^[S1068] TgB1F	5′- AGA GAC ACC GGA ATG CGA TCT -3′
^1^[S1069] TgB1R	5′- TTC GTC CAA GCC TCC GAC T -3′
^1^[S1077] TgB1P	5′SUN—TCG TGG TGA /ZEN/ TGG CGG AGA GAA TTG A—IABkFQ′3
Toxoplasma gondii REP assay	
^1^[S1070] TgREPF	5′- GAA AGC CAT GAG GCA CTC CA -3′
^1^[S1071] TgREPR	5′- TTC ACC CGG ACC GTT TAG C -3′
^1^[S1075] TgREPP	5′6-FAM—CGG GCG AGT /ZEN/ AGC ACC TGA GGA GAT ACA—IABkFQ′3
Neospora caninum NC5 assay	
^2^[S1064] NC5-550	5′- GGG TGA ACC GAG GGA GTT G -3′
^2^[S1065] NC5-596	5′- ACG TGA GGA ATG ACT AAC CAC AA -3′
^2^[S1078] NC5-P	5′SUN—AGC GGT GAG /ZEN/ AGG TGG GAT ACG TGG—IABkFQ′3
Hammondia hammondi Hham222	
^3^[S1066] Hham275F	5′- CTA CAA GGG GAG CGT CCT CG -3′
^3^[S1067] Hham81R	5′- GAG GAG AGT CGG AGA GGG AG -3′
^3^[S1076] Hham222P	5′6-FAM—TCC GGC TTC /ZEN/ AGT CTT TCC AC—IABkFQ′3
Mammalian GAPDH	
^4^[S1072] MAM_F	5′- TCA ACG GAT TTG GCC GTA TTG G -3′
^4^[S1073] MAM_R	5′- TGA AGG GGT CAT TGA TGG CG -3′
^4^[S1074] MAM_P	5′Cy5—CAG GGC TGC /TAO/ TTT TAA CTC TGG CAA AGT GGA -IAbRQSp3′

^1^adopted from Fekkar et al. (2008); ^2^ adopted from Ghalmi et al. (2008); ^3^ adopted from Schares et al. (2021); ^4^ adopted from Orr et al. (2020)

## Results and discussion

**Fig. 1:**
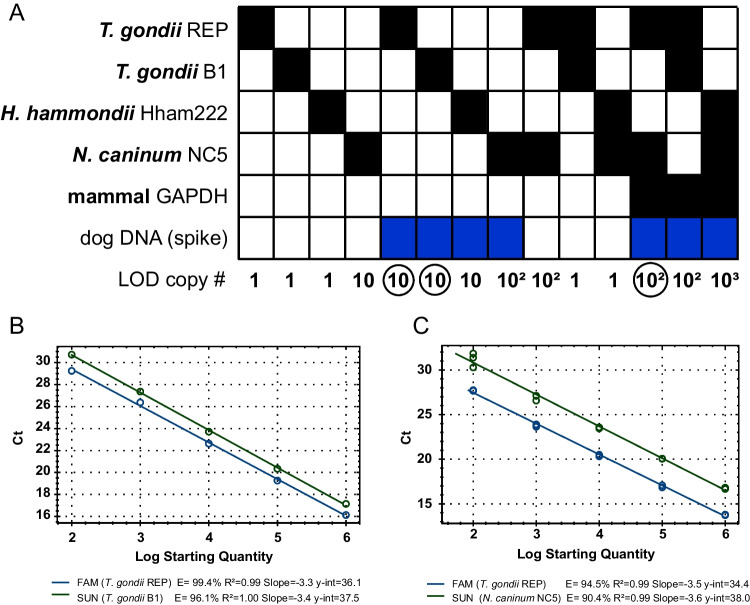
**A** Determination of limit of detection using synthetic DNA for cyst-forming coccidia. Probe-based assays to detect *Toxoplasma gondii* DNA, *Hammondia hammondi* DNA and *Neospora caninum* DNA were run independently or in combination with/or without host DNA (spike). The multiplexed assay was further combined with internal control assay detecting the target host—mammal, using generic GAPDH assay. Assays used are indicated by black squares and presence of host DNA by blue square. Limit of detection (LOD) in copy numbers (#) is depicted below the checkerboard. Encircled numbers represent assays expanded in **B** and **C** panels. **B** Standard curve for *T. gondii* assays targeting REP and B1 in the presence of mammalian DNA. **C** Standard curve for *T. gondii* assay targeting REP and *N. caninum* assay targeting NC5 region in the presence of mammalian DNA

Four probe-based qPCR assays targeting cyst-forming coccidia (*T. gondii*, *H. hammondi* and *N. caninum*) were combined with or without the host DNA To determine the limit of detection (LOD) in the form of copy numbers (Fig. 1A). For example, both *T. gondii* qPCRs assays targeting either REP or/and B1 were able to detect a single copy of a synthetic DNA without the presence of host DNA either independently or multiplexed (Fig. 1A). When host DNA was added to each assay the limit of detection was 10 copies for both REP and B1 targets. Multiplexing *T. gondii* REP and B1 assays with the mammalian assay further decreased LOD to 100 copies (Fig. 1A). Combination of the *H. hammondi* Hham222 proved to be difficult as the multiplexed assay failed to reach optimal *E* and *R*^2^. Mammalian DNA was detected and successfully amplified in all reactions and for all the above assays the efficacy and R^2^ values were acceptable (*E* = 90–110%, *R*^2^ > 0.99). When comparing the amplification curves for *T. gondii* assays, the REP qPCR amplified approximately one cycle earlier in the same dilutions of the target DNA compared the B1 qPCR assay (Fig. 1B). Similar to Galvani et al. (2019) and Barry et al. (2019), this study uses synthetic DNA rather than biological DNA in multiplexed real-time qPCRs for the detection of *T. gondii* and *N. caninum*. Our study not only quantified each assay’s LOD and efficiency, but also simulated realistic samples through the addition of mammalian DNA. This is a key point as the effects of background mammalian DNA on the diagnostic assay were previously unknown.

Taking the above results into an account, the *T. gondii* REP assay was selected with the *N. caninum* NC5 assay and combined with the host mammalian qPCR assay. Although a loss of LOD was observed compared to the single-plex assay, the multiplexed qPCR could detect and amplify 100 copies for both targets (*T. gondii* REP, *N. caninum* NC5) in all three replicates in the presence of host DNA (*E* = 90–110%, *R*^2^ > 0.94) (Fig. 1AC). The LOD for both is sufficient to detect a single organism (tachyzoite), because REP is present in 150–300 copies per *T. gondii* genome and NC5 is present in ~ 350 copies per genome of *N. caninum* (Okeoma et al. 2004). Attempts to add *H. hammondi* to the multiplexed qPCR were unsuccessful and such an assay will be required to be run separately as originally intended (Schares et al. 2021).

This study recorded a reduction in LODs when assays were combined. This issue has been noted in past literature where multiplexing was found to reduce assay analytical sensitivity due to primer interference and formation of nonspecific products (Choi et al. 2018; Peleg et al. 2010). Assuring consistent and reliable PCR performance is part of quality assurance in any diagnostic laboratory to deliver consistent, relevant, and accurate results (Bustin 2010). The present study took advantage of synthetic DNA targets to define analytical sensitivity as the smallest amount of the target measured in copy numbers in a sample that can accurately be measured. The analytical sensitivity expressed as LOD was used here to demonstrate the PCRs’ performance under a variety of conditions that the assays are intended for such as multiplexing in the presence of host DNA. This approach aligns with the Minimum Information for Publication of Quantitative Real-Time PCR Experiments (MIQE) (Bustin 2010). Determination of analytical sensitivity enables informed decision when combining assays. While loss of analytical sensitivity may not be the critical issue for *T. gondii* and *N. caninum* assay, multiplexing with *H. hammondi* and applying MIQE guidelines exposed underperformance of the assay both in terms of analytical sensitivity as well as assay efficacy. Our study confirmed that *T. gondii* REP qPCR assay is preferred as it amplifies the target earlier yet is equal as far as analytical sensitivity when compared to the *T. gondii* B1 assay (Belaz et al. 2015; Pomares et al. 2020).

To conclude, the *T. gondii* and *N. caninum* multiplexed qPCR assay optimised in this study can be used to effectively detect parasite DNA for diagnostic purposes in animal tissue.

## Data Availability

All data are included in the manuscript.
